# Prognosis and longitudinal changes of physical activity in idiopathic pulmonary fibrosis

**DOI:** 10.1186/s12890-017-0444-0

**Published:** 2017-07-25

**Authors:** Thomas Bahmer, Anne-Marie Kirsten, Benjamin Waschki, Klaus F. Rabe, Helgo Magnussen, Detlef Kirsten, Marco Gramm, Simone Hummler, Eva Brunnemer, Michael Kreuter, Henrik Watz

**Affiliations:** 1 LungenClinic Grosshansdorf, Pneumology, Woehrendamm 80, 22927 Grosshansdorf, Germany; 2 Pulmonary Research Institute at LungenClinic Grosshansdorf, Woehrendamm 80, 22927 Grosshansdorf, Germany; 3 Center for Interstitial and Rare Lung Diseases, Pneumology, Thoraxklinik, University of Heidelberg, Röntgenstrasse 1, 69126 Heidelberg, Germany; 4 Airway Research Center North (ARCN), German Center for Lung Research (DZL), Grosshansdorf, Germany; 5 Translational Lung Research Center Heidelberg (TLRC), German Center for Lung Research (DZL), Heidelberg, Germany

**Keywords:** Functional status (activity levels), Physical exercise, Triaxial accelerometer, Mortality, Longitudinal studies, Idiopathic pulmonary fibrosis

## Abstract

**Background:**

Physical activity (PA) is associated with disease severity in idiopathic pulmonary fibrosis (IPF), but longitudinal studies evaluating its prognostic value and changes over time are lacking.

**Methods:**

We measured PA (steps per day, SPD) in a cohort of 46 IPF-patients (mean age, 67 years; mean FVC, 76.1%pred.) by accelerometry at baseline, recorded survival status during 3 years follow-up and repeated measurements in survivors. We compared the prognostic value of PA to established mortality predictors including lung function (FVC, DLCO) and 6-min walking-distance (6MWD).

**Results:**

During follow-up (median 34 months) 20 patients (43%) died. SPD and FVC best identified non-survivors (AUROC-curve 0.79, *p* < 0.01). After adjustment for confounders (sex, age, therapy), a standardized increase (i.e. one SD) in SPD, FVC%pred. or DLCO%pred. was associated with a more than halved risk of death (HR < 0.50; *p* < 0.01). Compared to baseline, SPD, FVC, and 6MWD annually declined in survivors by 973 SPD, 130 ml and 9 m, resulting in relative declines of 48.3% (*p* < 0.001), 13.3% (*p* < 0.001) and 7.8% (*p* = 0.055), respectively.

**Conclusion:**

While PA predicts mortality of IPF patients similar to established functional measures, longitudinal decline of PA seems to be disproportionally large. Our data suggest that the clinical impact of disease progression could be underestimated by established functional measures.

## Background

Idiopathic pulmonary fibrosis (IPF) is a progressive fibrosing interstitial pneumonia with poor prognosis [1]. Despite successful development of new therapies, survival has not been convincingly improved [1–3]. Identification of validated predictors of disease progression and mortality in IPF is essentially important for decision making in clinical practice. Established mortality predictors have been derived from large cohorts of IPF patients, and fundamental baseline characteristics such as gender, age, lung function, exercise capacity, and comorbidities have demonstrated significant potential [4–12].

Lung function decline is frequently chosen as outcome measure in clinical trials, serving as a marker for disease progression and mortality [2, 3]. However, validity and clinical meaning of this measure is controversial [13–18]. A recent expert working group report suggested that besides primary survival analyses, the best evidence of the clinical efficacy of a treatment might be derived from direct measures of a patient’s symptoms and daily functions [13]. Therefore, measures of daily physical activity (PA) might represent a clinically meaningful surrogate of a patient’s well-being and everyday functional status [13, 19]. Indeed, close relationships can be shown between PA and patient-reported outcomes, such as dyspnea, fatigue, and health status in patients with IPF [20]. However, to the best of our knowledge there are currently no studies available that have measured PA in IPF longitudinally. Mortality and objectively measured physical activity have previously shown some associations in patients with various entities of fibrotic idiopathic interstitial pneumonia [21], but data on patients with established IPF are lacking. Furthermore, there is no detailed analysis available comparing the prognostic value of physical activity to established clinical indicators of disease severity and progression, such as lung function and exercise capacity.

In our study, we aimed to investigate (1) the predictive value of physical activity for all-cause mortality and (2) the amount of physical activity decline in a prospective longitudinal cohort of patients with IPF at two tertiary centers for Interstitial Lung Diseases in Germany.

## Methods

### Study population

In 2013, we included 48 patients with IPF (36 men, 12 women) in this prospective observational cohort and performed follow-up visits between June 2016 and October 2016. All visits took place at the Pulmonary Research Institute at LungenClinic Grosshansdorf, Grosshansdorf, Germany, and at the Center for Interstitial and Rare Lung Diseases, Thoraxklinik, University Heidelberg, Heidelberg, Germany. Cross-sectional analyses at baseline and detailed methods have been published previously [20]. Briefly, study participants were initially recruited in the outpatient departments of LungenClinic Grosshansdorf, and Thoraxklinik Heidelberg, respectively, and diagnosis of IPF at each center was established in an interdisciplinary discussion (Interstitial Lung Disease Board), according to current guidelines [1].

We contacted the patients 3 years after the baseline visit by telephone and invited them for a follow-up visit at the study centers. Exact dates of death from deceased patients were obtained from institutional databases, relatives or treating physicians. Follow-up visits in survivors followed the same protocol as the baseline visits regarding the assessment of lung function, exercise capacity, physical activity, and medical history. Two patients were too ill to attend the outpatient departments. In these two cases we performed a telephone interview, asked the patient to send us the latest available lung function report, and supplied them with an activity monitor via postal service. Patients who underwent lung transplantation within the observation period (*n* = 2) were excluded from this study. Censor date for mortality analyses was October 4th 2016. Baseline and follow-up visits were performed at least 4 weeks after the latest respiratory tract infection or exacerbation.

The study was approved by the Ethics Committees of the Medical Association Schleswig-Holstein (AZ 038/12 II), and of the University of Heidelberg (S-200/2013), respectively. All participants provided written informed consent.

### Physical activity

We measured physical activity using the same multisensory armband (SenseWear Pro; BodyMedia Inc., Pittsburgh, PA, USA) at baseline and at follow-up over a period of 1 week each, as previously described [20, 22]. The armband is worn on the upper left arm over the triceps muscle and incorporates amongst others a triaxial accelerometer that records average steps per day, which correlate best with dyspnea, quality of life and lung function in patients with IPF [20]. We instructed patients to wear the armband for 24 h/ day except for the time spent on personal hygiene. A threshold of 94% of wearing time (22.5 h) was used to identify valid days [22].

### Lung function and exercise capacity

We performed spirometry (forced vital capacity, FVC; forced expiratory volume at one second, FEV_1_) and diffusing capacity for carbon monoxide (DLCO) in line with current guidelines using established reference values [23, 24]. We measured the 6-min walking distance (6MWD) according to previous guidelines [25].

### Disease severity and disease progression

We assessed disease severity and risk of mortality according to the GAP (Gender – Age – Physiology) score and GAP staging system [8]. We defined stable IPF as FVC %pred. decline less than 5% [26], and 6MWD decline less than 30 m [27] according to established values for minimal clinically important differences (MCID) in IPF.

### Statistical analysis

Descriptive data of survivors and non-survivors are reported as mean and standard deviation (continuous variables), median and inter-quartile range (categorical variables and continuous variables with skewed distribution), or number and percent (dichotomous variables), as appropriate. Cross-sectional differences were compared using two-tailed t-test for normally distributed variables, Mann-Whitney-U test for categorical variables, and χ^2^ tests for dichotomous variables.

We analyzed the relationship of physical activity and all-cause mortality by Kaplan-Meier survival plots and log rank tests using tertiles of steps per day. We compared these findings to measures of lung function (i.e. FVC %pred. and DLCO %pred.) and exercise capacity (i.e. 6MWD), and therefore these variables were also classified according to tertile distribution. We then calculated the area under the receiver operating characteristic (AUROC) curve for the different continuous variables and for the GAP score, respectively, to discriminate between survivors and non-survivors. The results of AUROC-curve analyses can be interpreted in analogy to C-statistics. In a next step, we used multivariate COX regression analyses to estimate the relative risk of all-cause mortality associated with the different variables. First, we calculated the crude hazard ratios for each raw parameter. Second, we standardized variables for better comparability by dividing continuous variables by one standard deviation. These standardized variables then were introduced in a multivariate COX regression model that was also adjusted for sex, age, and antifibrotic therapy as basic confounders. This second step was not done for the GAP score that already includes age and sex. In a final step, we then investigated whether physical activity had any additional effect on mortality prediction beyond lung function (i.e. FVC %pred. and DLCO %pred., respectively).

In survivors, longitudinal differences between measurements at baseline and follow-up were tested by paired t-test or Wilcoxon rank, as appropriate. Average annual decline rates among 3-year survivors were calculated by referring the absolute decline rates to a fixed time interval, i.e. follow-up time in years ((follow-up value – baseline value)/ years to follow-up). Among patients with complete baseline and follow-up data for physical activity, we identified the proportion of patients with stable disease throughout the observation period, according to pre-specified decline rates for lung function and exercise capacity, respectively. Finally, we compared the amount of physical activity decline between patients with stable disease and progressive disease with separate ANOVAs for repeated measures, respectively.

A *p*-value <0.05 was considered statistically significant. Data analysis was performed with the Statistical Package for Social Science, version 20.0 (SPSS, Chicago, Ill. USA).

## Results

We followed 46 patients with IPF over a median time of 34 [20–38] months. Twenty patients (43%) died within this observation period. At baseline, non-survivors had lower lung function, exercise capacity and physical activity compared to survivors (Table 1). Furthermore, non-survivors had a higher GAP score and used antifibrotic therapy less frequently (Table 1). Frequencies of comorbidities were numerically different for some disease entities (Table 1). Among survivors, the type of antifibrotic drug was unchanged throughout the observation period in 19 patients (73%), and changed in five patients (19%); in one patient (4%) antifibrotic therapy was completely stopped, and in one other patient (4%) newly initiated.

**Table 1:** Patient characteristics at baseline by survival status

Characteristic	Survivors (*n* = 26)	Non-survivors (*n* = 20)	*p*-value
Description			
Age, years	65.8 (6.8)	69.2 (8.5)	0.14
Male	18 (69%)	17 (85%)	0.21
BMI, kg/m^2^	28.2 (4.0)	27.7 (4.9)	0.67
Use of Antifibrotic Therapy	24 (92%)	14 (70%)	0.048
LTOT	5 (20%)	5 (25%)	0.69
pO_2_ at rest, mmHg	72.1 (8.2)	70.2 (9.7)	0.50
Lung Function			
FVC, liter	3.1 (0.8)	2.3 (0.7)	0.002
FVC, %pred.	85.1 (21.4)	64.3 (17.2)	0.001
FEV_1_ / FVC	0.85 (0.06)	0.81 (0.07)	0.08
DLCO, %pred.	48.7 (13.5)	35.9 (12.9)	0.002
Exercise Capacity			
6MWD, meters	415 (128)	299 (118)	0.003
Physical Activity			
Steps per day	6606 (3064)	3433 (2655)	+0.001
Disease Severity			
GAP Score	4 [2–4]	5 [4–5]	0.005
GAP Stage	II [I – II]	II [II – II]	0.02
Comorbidities			
Pulmonary Hypertension	1 (4%)	1 (5%)	0.85
Arterial Hypertension	9 (35%)	10 (50%)	0.29
Cardiovascular Diseases^a^	7 (27%)	7 (35%)	0.56
Diabetes Mellitus	1 (4%)	4 (20%)	0.08
GERD	6 (23%)	2 (10%)	0.25
Lung Cancer	0	1 (4%)	0.25
Depression	1 (4%)	0	0.38
Mean number of comorbidities per patient ≥ 1^b^	17 (65%)	17 (85%)	0.13

Data are presented as mean (SD), median [IQR] or n (%) depending on scale and distributional characteristics

Data were missing for LTOT (*n* = 1) and pO2 (*n* = 2)

*Abbreviations*: *LTOT* long-term oxygen therapy, *pO*_*2*_ partial pressure of oxygen in capillary blood analysis, *FVC* forced vital capacity, *FEV*_*1*_ forced expiratory volume at one second, *DLCO* diffusing capacity of the lung for carbon monoxide, *6MWD* 6-min walking-distance, *GAP* Gender-Age-Physiology, *GERD* gastro-esophageal reflux disease

^a^Cardiovascular diseases encompassed coronary artery disease, left heart failure or any other cardiovascular diseases, respectively

^b^ *N* = 9 (35%) and *n* = 3 (15%) patients had no comorbidities, respectively. Nobody had ≥4 comorbidities

**Fig. 1:**
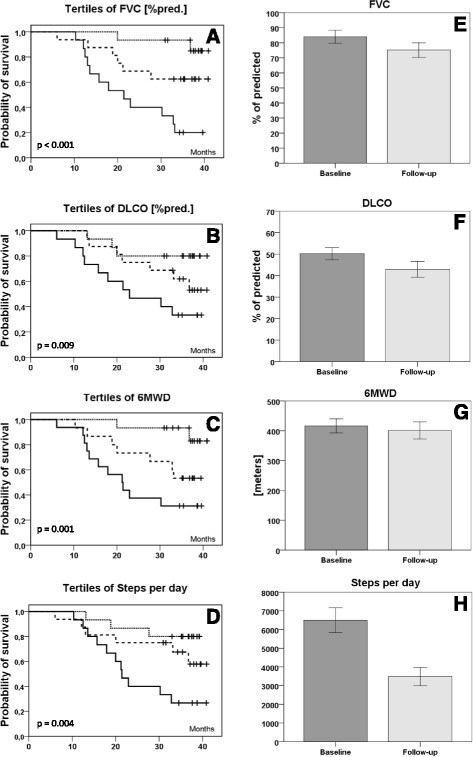
Kaplan-Meier survival curves for tertiles of baseline values of forced vital capacity (FVC %pred.) (**a**), diffusing capacity of the lung for carbon monoxide (DLCO %pred.) (**b**), 6-min walking distance (6MWD) (**c**) and steps per day (**d**) are displayed on the left. Longitudinal changes (i.e. baseline and follow-up values of survivors) of FVC %pred. (**e**), DLCO %pred. (**f**), 6MWD (**g**) and steps per day (**h**) are displayed as bar-charts on the *right*. In Kaplan-Meier survival curves solid lines represent the lowest tertile, *dashed lines* the mid tertile and *dotted lines* the highest tertile of the baseline values of FVC %pred. (**a**), DLCO %pred.(**b**), 6MWD (**c**) and steps per day (**d**), respectively. Censored data of surviving patients are represented as vertical dashes. *P*-values are presented within the single graphs. *Bar-charts* represent baseline (*dark grey, left*) and follow-up (*light grey, right*) data of FVC %pred. (**e**), DLCO %pred. (**f**), 6MWD (**g**) and steps per day (**h**). Comparissons between baseline and follow-up were made with paired t-tests and *p*-values were <0.001, 0.024, 0.055 and <0.001, respectively

The probability of surviving the observation period differed significantly across tertiles of FVC %pred., DLCO %pred., 6MWD, and steps per day (Fig. 1a-d).

**Fig. 2:**
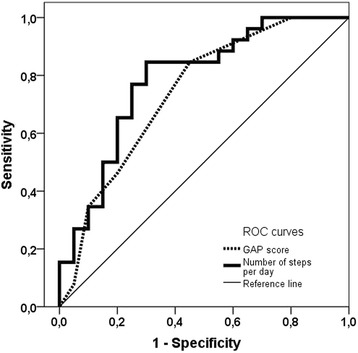
Receiver operating characteristic (ROC) curves for the GAP score and the number of steps per day. The corresponding areas under the curves (AUROC) were 0.74 and 0.79, respectively

The prognostic values of lung function, exercise capacity, steps per day, and GAP score for mortality prediction are shown in Table 2. Each variable was significantly associated with an increased risk of death in patients with IPF (Table 2). Steps per day and FVC %pred. showed the best ability (i.e. sensitivity and specificity) to distinguish between survivors and non-survivors with an AUROC curve of 0.79 (Table 2; Fig. 2). Adjusting for sex, age and therapy, and using standardized continuous variables, a standardized increase (i.e. one SD) in steps per day, FVC %pred. or DLCO %pred. was associated with a more than halved risk of death (HR < 0.50; *p* < 0.01) (Table 2). In multivariate Cox regression analyses adjusting for age, sex, and either FVC %pred. or DLCO %pred., steps per day showed no independent additional effect on mortality prediction (HR 0.99988 [0.99970–1.00007], *p* = 0.216; HR 0.99989 [0.99970–1.00007], *p* = 0.225; respectively).

**Table 2:** Prognostic value of physical activity and established predictors of mortality in patients with IPF

	C statistic			Crude Cox regression of the raw predictors			Adjusted Cox regression of the standardized predictors		
	AUROC	95% CI	*p*-value	HR	95% CI	*p*-value	HR	95% CI	*p*-value
Lung Function									
FVC %pred.	0.79	0.65–0.92	0.001	0.96	0.94–0.99	0.001	0.36	0.20–0.66	0.001
DLCO %pred.	0.78	0.64–0.92	0.001	0.94	0.91–0.98	0.002	0.33	0.17–0.67	0.002
Exercise Capacity									
6MWD	0.77	0.63–0.91	0.002	0.96^a^	0.93-0.98	0.003	0.56	0.37–0.87	0.009
Physical Activity									
Steps per day	0.79	0.65–0.92	0.001	0.97^b^	0.96-0.99	0.002	0.46	0.26–0.82	0.008
Disease Severity									
GAP Score	0.74	0.59–0.89	0.006	1.63	1.19–2.24	0.003	n/a	n/a	n/a

Adjustment was done for sex, age and antifibrotic therapy. For standardization, all continuous variables were divided by one standard deviation

*Abbreviations*: *AUROC* area under the receiver operating characteristics, *HR* hazard ratio, *CI* confidence interval, *FVC* forced vital capacity, *DLCO* diffusing capacity of the lung for carbon monoxide, *6MWD* 6-min walking-distance, *GAP* Gender-Age-Physiology

^a^Crude HR for 6MWD is reported per 10 m change. Therefore 6MWD results were divided by 10

^b^Crude HR for SPD is reported per 100 steps change. Therefore SPD results were divided by 100

In patients surviving the observation period (*n* = 26), valid physical activity data were available in 23 cases at follow-up. In two patients wearing time of the accelerometer did not meet predefined criteria, and one patient refused to repeat the PA measurement at follow-up. Among survivors with complete follow-up data for physical activity, lung function and physical activity were significantly lower at follow-up compared to baseline, while exercise capacity as measured by 6MWD showed a statistic trend only (Fig. 1e-h, Table 3). Relative declines between baseline and follow-up were disproportionally larger for physical activity compared to lung function and 6MWD (Table 3). According to pre-specified decline rates for FVC%pred. and 6MWD (i.e. FVC%pred. <5%, and 6MWD <30 m), 39% (*n* = 9) and 48% (*n* = 11) of the patients with complete data sets were classified as having stable disease, as longitudinal changes after 3 years were below the MCID, respectively. Among patients with stable FVC and 6MWD, physical activity declined by 3213 ± 2203 and 3198 ± 2812 steps per day, respectively. Patients with progressive disease - as indicated by FVC and 6MWD decline - showed similar physical activity decline (3033 ± 2594 and 3120 ± 2090 steps per day, respectively; *p*-values 0.414 and 0.264, respectively).

**Table 3:** Longitudinal changes in lung function, exercise capacity and physical activity in survivors

Characteristic	Absolute value at baseline	Absolute value at follow-up	Absolute decline (Baseline to follow-up)	Relative decline (Baseline to follow-up)	Average annual decline	*p*-value
FVC, liter	3.05 (0.81)	2.64 (0.78)	−0.41 (0.33)	13.3%	−0.13 (0.11)	<0.001
FVC, %pred.	84.8 (22.9)	76.1 (23.9)	−8.7 (9.6)	10.5%	−2.8 (3.0)	<0.001
DLCO, %pred.	50.7 (13.6)	43.5 (18.4)	−7.3 (13.3)	14.4%	−2.2 (4.2)	0.024
6MWD, meters	418 (117)	389 (132)	−29 (64)	7.8%	−8.8 (20)	0.055
Steps per day	6499 (3192)	3482 (2350)	−3017 (2372)	48.3%	−973 (743)	<0.001

Data are presented as mean (SD) and % for *n* = 23 survivors with complete physical activity data at follow-up. Differences between baseline and follow-up visit were tested by paired t-tests. Follow-up data were missing for FVC (*n* = 1), DLCO (*n* = 3), and 6MWD (*n* = 2)

*Abbreviations*: *FVC* forced vital capacity, *DLCO* diffusing capacity of the lung for carbon monoxide, *6MWD* 6-min walking-distance

## Discussion

The main finding of our study is that objectively measured physical activity is a novel predictor of mortality in patients with IPF. Furthermore, physical activity nearly halves in IPF patients surviving a follow-up period of 3 years. The ability to predict mortality is similar to established measures such as lung function, while physical activity decline in survivors is much more pronounced than suggested by longitudinal changes in lung function or 6MWD only.

Physical activity has just recently been investigated in patients with IPF in cross-sectional studies, and associations with lung function, exercise capacity and patient-reported outcomes have been demonstrated [20, 21, 28, 29]. Our study is the first to present longitudinal data. In patients with IPF we observed a 48% decline in steps per day within the observation period of 3 years. Interestingly, after these 3 years with an average annual decline of nearly 1000 steps per day the total number of steps in survivors very much resembled the initial number of steps in patients that didn’t survive this observation period. Furthermore, the physical activity decline in IPF seems to be more than twice as high compared to the changes in patients with severe COPD, in whom a previous study found average annual decline rates of 435 and 461 steps in COPD patients with GOLD III and IV, respectively [30].

Compared to the large decline in physical activity in patients with IPF, decline in exercise capacity was disproportionally smaller. Generally, those patients that survived the observation period showed a rather preserved exercise capacity at baseline and follow-up. However, the observation period of 3 years is rather long for a study with IPF patients and coincides with the median survival time [1]. Therefore, the relatively preserved exercise capacity in our study might be biased by the fact that patients with rapid 6MWD-decline did not survive three years of observation [10]. For that same reason, the data on 6MWD-decline from other cohorts with an observation period of twelve months only, might not be comparable to our 3-year data with its calculated annual decline [27, 31, 32]. Nevertheless, our present study depicts an astonishing discrepancy between 6MWD and physical activity when studied longitudinally in patients with IPF. This discrepancy suggests that physical activity and exercise capacity reflect different aspects of the functional status of IPF patients when the disease progresses.

FVC currently is the most frequently chosen measure of disease progression in IPF and standard primary outcome in clinical studies [2, 3]. In recent phase 3 clinical trials antifibrotic agents have successfully reduced FVC-decline and slowed down disease progression, as indicated by the proportion of patients with an FVC-decline below 10% [2, 3]. However, conclusions with respect to mortality and the functional status of the patients remain a matter of debate [2, 3, 13–15]. In our study, average annual FVC-decline of 130 ml is in line with recent clinical trial results of pirfenidone [2] and nintedanib [3], given the fact that all but two of the surviving IPF patients received either one of the two treatments. According to FVC-decline, up to 40% of the surviving patients would have been evaluated as having stable disease - even when choosing a more conservative threshold (i.e. relative FVC-decline <5%) [26]. However, physical activity decreased in both patients with relatively stable FVC and in those patients with a clinically meaningful decline in FVC. Interestingly, decline rates were similar between both groups. These results suggest that longitudinal measures of FVC might underestimate clinical deteriorations as indicated by physical activity decline.

Although numbers are small and only two time points are available, we believe that our results support further evaluation of physical activity as an objective measure of disease progression in IPF. Measures of physical activity represent a direct objective measure of a patient’s functional status, and when assessed longitudinally they reflect information beyond currently established surrogates of disease progression. Complementary assessment of both physical activity and established clinical variables might improve adequate prediction of the course of the disease, as measures of lung function decline alone rather poorly predicted future disease progression [32].

Previous studies showed a link between activities of daily living or self-reported daily physical activity and mortality in patients with IPF [33, 34]. However, questionnaire data need to be interpreted with care as the amount of physical activity might be under- or overestimated by the patients [19]. Furthermore, they are subject to recall bias and require confirmation by objective measurements, especially when studied in smaller patient cohorts [19]. Objectively measured physical activity and mortality have also been linked to another in a previous study by Wallaert et al. in patients with various entities of fibrotic idiopathic interstitial pneumonia during a follow-up period ranging from 6 to 36 months [21]. The various fibrotic entities and wide range of follow-up makes it difficult to draw firm conclusions for patients with IPF. Nevertheless, in the study by Wallaert et al. 3300 steps per day were associated with an increased risk of mortality, which is very similar to the baseline count of 3400 steps per day in non-surviving IPF patients in our study [21].

Comparing physical activity to established mortality predictors, such as lung function we found very similar prognostic capacity. Furthermore, we saw no independent contribution of physical inactivity on mortality prediction beyond lung function impairment in this study. By contrast, we showed previously in patients with COPD that physical activity is the strongest predictor of all-cause mortality with a substantial prognostic capacity beyond established predictors like lung function or exercise capacity [35].

Objective and valid surrogates of mortality in patients with IPF are an urgent clinical need, as therapeutic trials studying all-cause mortality in IPF may not be feasible, due to the necessary size, duration and cost [17]. The results of our study suggest that objectively measured daily physical activity might be a valuable surrogate of mortality in IPF that deserves further evaluation in large cohorts of IPF patients, and that might possibly also be considered as complementary outcome measure in clinical trials.

Our study has several limitations. First, the sample size is relatively small which might lead to either over- or underestimation of certain effects. Therefore, larger studies are needed to confirm our findings. Second, survivors used antifibrotic therapy more frequently and tended to be younger. As this is an observational study we cannot exclude that both confounding factors affected our results, even though we adjusted for these confounders. Third, there are only two time points available. Application of average decline rates in longitudinal studies with only two measurements presumes that the disease course is linear, which might not be true for IPF [32].

## Conclusion

Our data suggest that in patients with IPF objectively measured daily physical activity might be a valuable assessment for disease progression and mortality, respectively. Current measures of disease progression might not adequately reflect the impact of clinical worsening of the disease on the patient’s everyday life.

## Abbreviations

6MWD: 6-Minute walking distance
COPD: Chronic obstructive pulmonary disease
DLCO: Diffusing capacity of the lung for carbon monoxide
FEV_1_: Forced expiratory volume at one second
FVC: Forced vital capacity
ILD: Interstitial lung disease
IPF: Idiopathic pulmonary fibrosis
LTOT: Long-term oxygen therapy
PA: Physical activity
pO_2_: Partial pressure of oxygen in capillary blood analysis
SPD: Steps per day
SPSS: Statistical Package for Social Science
